# Hydroxytryptamine transporter gene-linked polymorphic region (5HTTLPR) is associated with delusions in Alzheimer’s disease

**DOI:** 10.1186/s40035-019-0144-1

**Published:** 2019-02-01

**Authors:** Grazia D’Onofrio, Francesco Panza, Daniele Sancarlo, Michele Lauriola, Mariangela P. Dagostino, Giulia Paroni, Madia Lozupone, Antonio Mangiacotti, Paola Bisceglia, Carolina Gravina, Maria Urbano, Filomena Addante, Francesco Paris, Leandro Cascavilla, Antonio Greco, Davide Seripa

**Affiliations:** 1 Fondazione Casa Sollievo della Sofferenza, Department of Medical Sciences, Geriatrics Unit, San Giovanni Rotondo (FG) Foggia, Italy; 2 Neurodegenerative Disease Unit, Department of Basic Medicine, Neuroscience, and Sense Organs, University of Bari Aldo Moro, Bari, Italy; 3 Department of Clinical Research in Neurology, University of Bari Aldo Moro, Pia Fondazione Cardinale G. Panico, Tricase, Lecce, Italy

**Keywords:** Delusions, Neuropsychiatric inventory, Alzheimer’s disease, 5-HTTLPR, Serotonin

## Abstract

**Background:**

Serotoninergic pathways underlying delusion symptoms in Alzheimer’s disease (AD) have not been fully clarified. 5-Hydroxytryptamine transporter gene-linked polymorphic region (5-HTTLPR) is a variable number tandem repeats in the promoter region of serotonin transporter encoding-gene affecting transcription.

**Methods:**

We investigated the association of 5-HTTLPR with delusions in a total of 257 consecutive patients clinically diagnosed as AD according to the National Institute on Aging-Alzheimer’s Association criteria. All participants underwent a comprehensive evaluation with a standardized comprehensive geriatric assessment and Neuropsychiatric Inventory.

**Results:**

Delusion symptoms were observed in 171 patients (66.54%). In respect to AD patients without delusions, AD patients with delusions showed a low prevalence of S-plus carriers (5-HTTLPR-L/S + 5-HTTLPR-S/S genotypes) [*p* < 0.001; odds ratio (OR) = 0.240, 95% confidence interval (CI) = 0.121–0.471]. Logistic regression analysis adjusted for the apolipoprotein E polymorphism showed that in AD patients with delusions the presence of an 5-HTTLPR-S allele may reduce disease duration (*p* = 0.005; OR = 0.680, 95% CI = 0.522–0.886) and increase aberrant motor activity (*p* = 0.013; OR = 2.257, 95% CI = 1.195–4.260). The present findings suggested that 5-HTTLPR might be associated with delusions in AD. S-plus carriers might be associated with protective effect against delusions in AD.

**Conclusions:**

More studies on wider samples of high selected demented patients are needed to confirm our results. However, the present findings suggested that a genetic factor related to serotonin metabolism might exert a protective role on the clinical expression of neuropsychiatric clusters in AD with important implications regarding mechanisms underlying delusions and their possible treatment across the AD and dementia spectrum.

**Electronic supplementary material:**

The online version of this article (10.1186/s40035-019-0144-1) contains supplementary material, which is available to authorized users.

## Background

Alzheimer’s disease (AD) accounts for the 60–70% of cases of dementia. Current estimates indicate 17% of people aged 75–84 years in the US have AD, and the disease costs the country $277 billion per year [1]. Dementia costs in Italy were estimated to be €39.3 billion in 2016 [2]. The prevalence is projected to triple by 2050 to 13.8 million with annual costs of >$1.1 trillion [1]. AD is often complicated by neuropsychiatric symptoms (NPS), which occur in one-third of patients at an early stage of the disease [3, 4]. The possible hypotheses for psychopathology in AD involve selective loss of different neuronal populations, alterations of specific neurotransmitter systems, and genetic risk factors [5, 6]. Delusions appearing with their disabling and persistent features is among the most common NPS characterizing AD course and ranging from 10 to 73% [7]. In a recent study, as compared with AD patients without delusions, it was shown that AD patients with delusion showed higher dementia severity, and higher impairment in cognitive and depressive symptoms, and in several neuropsychiatric domains, and this appeared to be associated with higher multidimensional impairment and increased risk of mortality [8].

Previous studies have shown that NPS in AD were highly heritable [9], increased AD familial risk, and showed significant genome-wide linkage [10], providing evidence that genetic variation does contribute to delusion risk. More rapid cognitive decline was the most consistent correlate of AD with psychosis (AD+P), defined as the occurrence of delusions or hallucinations, compared to AD without psychosis (AD-P) [11]. A recent genome-wide association study (GWAS) showed for the first time an association of AD+P with polygenic risk for a unique set of common variants and inversely associated with polygenic risk for schizophrenia [12]. In this large GWAS, the biological pathways identified by the associations of schizophrenia single nucleotide polymorphisms (SNPs) with AD+P were endosomal trafficking, autophagy, and calcium channel signalling [12].

Investigations of loci in serotonergic gene pathways are among the best studied in association studies of AD with NPS [13, 14]. The 5-hydroxytryptamine (5-HT, serotonin) transporter (5-HTT) gene-linked polymorphic region (5-HTTLPR) is a well-characterized variable number of tandem repeat in the promoter region of the serotonin transporter gene, with two major alleles termed long (L) and short (S), suggesting to affect gene transcription [15, 16]. A deeper knowledge of the “5-HTTLPR universe” will be useful to better understand the molecular basis of serotonin homeostasis and the pathological basis underlying serotonin-related NPS conditions and traits.

The association of 5-HTTLPR with delusions in AD was studied in only few studies from Caucasian [17–21] and Japanese patients [22, 23], with contrasting evidence of association. In a recent study, the 5-HTTLPR was associated with delusions in dementia with Lewy bodies (DLB), with important implications regarding the mechanisms underlying this symptom across the AD/DLB/Parkinson disease (PD)-dementia spectrum [24]. The aim of the present study was to determine whether the 5HTTLPR serotonin transporter gene polymorphism was associated with delusions in patients with AD and to determine the association of 5-HTTLPR polymorphisms with other NPS.

## Methods

### Subjects

This prospective observational study fulfilled the Declaration of Helsinki [25], and the guidelines for Good Clinical Practice [26]. The approval of the study for experiments using human subjects was obtained from the local Ethics Committees on human experimentation. Written informed consent for research was obtained from each patient or from relatives/legal guardian in the case of critically disabled demented patients prior to participation in the study. To avoid bias due to different genetic backgrounds in the population, all patients and subjects included in this study were Caucasians and we did not include people of Jewish, Eastern Europe or Northern Africa descent, with most individuals at least two or more generation living in Central and Southern Italy for at least three generations. Patients consecutively evaluated from March 1, 2015 to December 31, 2016 at the Alzheimer’s Evaluation Unit of the Complex Structure of Geriatrics of the IRCCS “Casa Sollievo della Sofferenza”, San Giovanni Rotondo, Foggia, Italy were screened for eligibility.

### Inclusion/exclusion criteria

Inclusion criteria were: 1) age ≥ 65 years, 2) ability to provide an informed consent or availability of a relatives or a legal guardian in the case of severe demented patients, 3) diagnosis of AD according to the National Institute on Aging-Alzheimer’s Association (NIA-AA) criteria [27], 4) a complete cognitive and neuropsychiatric assessment, 5) a complete Comprehensive Geriatric Assessment (CGA) [28]. Exclusion criteria were: 1) diagnosis of non-AD dementia; 2) presence of serious comorbidity, tumors and other diseases that could be causally related to cognitive impairment (ascertained blood infections, vitamin B_12_ deficiency, anemia, disorders of the thyroid, kidneys or liver); 3) history of alcohol or drug abuse, head trauma, and other causes that can cause memory impairment; 4) a history of schizophrenia, schizoaffective disorder, delusional disorder or mood disorder with psychotic features, major depressive disorder, substance use disorder, or mental retardation according to Diagnostic and Statistical Manual of Mental Disorders - Fifth Edition (DMS-5) criteria [29].

### Comprehensive geriatric assessment

The CGA [28] was carried out using assessment instruments widely employed in geriatric practice. Functional status was evaluated by activities of daily living (ADL) index [30], and by instrumental activities of daily living (IADL) scale [31]. Cognitive status was screened by the Short Portable Mental Status Questionnaire (SPMSQ) [32]. Comorbidity was examined using the Cumulative Illness Rating Scale Comorbidity Index (CIRS-CI) [33]. Nutritional status was explored with the Mini Nutritional Assessment (MNA) [34]. The Exton-Smith Scale (ESS) [35] was used to evaluate the risk of developing pressure sores. Medication use was defined according to the Anatomical Therapeutics Chemical Classification code system, and the number of drugs used by patients was recorded. Social aspects included household composition, home service, and institutionalization.

### Cognitive assessment

In all patients, cognitive status was screened with the Mini-Mental State Examination (MMSE) [36]. Dementia was diagnosed following the DMS-5 criteria [29]. Diagnoses of possible/probable AD were made according to the NIA-AA criteria [27]. Diagnoses of possible/probable VaD were made according to the criteria of the National Institute of Neurological Disorders and Stroke - Association Internationale pour la Recherche et l’Enseignement en Neurosciences Work Group (NINDS-AIREN) [37]. In unclear cases, differential diagnosis between AD and VaD was based also on the Hachinski Ischemic Score: scores ≤4 were considered as probable AD, scores ≥7 were diagnosed as VaD [38]. Diagnosis of AD and VaD was always supported by neuroimaging evidence (computerised tomography scan and/or nuclear magnetic resonance). In particular, the presence of multiple cortical/subcortical infarcts or an infarct in a strategic area such as the thalamus or temporal lobe and/or lesions of the white matter indicated probable VaD; the absence of the above-mentioned cerebrovascular lesions indicated AD. The age at onset of AD symptoms was estimated by semi-structured interviews with the patients’ caregivers [39], body mass index(Kg/m2), disease duration, and delusion symptom duration were also calculated for AD patients.

### Neuropsychiatric assessment

Neuropsychiatric assessment was performed using the Neuropsychiatric Inventory (NPI) that is based on a structured interview with a caregiver and/or patient’s relative [40]. The following 12 neuropsychiatric domains were evaluated: delusions, hallucinations, agitation/aggression, depression mood, anxiety, euphoria, apathy, disinhibition, irritability/lability, aberrant motor activity, sleep disturbances, and eating disorders. For each domain, a screening question is asked to determine if the behavioral change is present or absent. If the answer is positive the domain is explored at greater depth with the sub-questions. If the sub-questions confirm the screening question, frequency is rated from 1 to 4 and severity is scored from 1 to 3. The product (severity x frequency) is calculated for each behavioural change present during the previous month or since the last evaluation. Patients with NPS were identified on the basis of the following parameters: presence of any delusions or hallucinations on the NPI (i.e., a score of ≥1 on either subscale), and/or dysphoria score > 6, anxiety score > 6, disinhibition score > 4, irritability/lability score > 2, and/or score on the apathy, agitation/aggression, euphoria, aberrant motor behavior, sleep disturbance, and eating disorder subscale > 1. In the present study, we used data from individual NPI delusions or hallucinations domains as binary data 0/1 for the definition of the AD-delusion subtype. Thus, a disturbance in the given domain was labelled as 1 = present if there was a score > 0 for hallucinations or delusions domain.

### Genetic analysis

Genomic DNA was purified from fresh/frozen blood samples following salting-out method [41]. Analysis of the 5-HTTLPR and apolipoprotein E (APOE) polymorphisms were made in blinded fashion previously described [42, 43].

### Statistical analysis

Normal distribution of continuous variables was verified by the Shapiro-Wilk normality test. Comparisons between continuous variables were performed by using the Welch Two Sample t-test (Parametric distribution) or the Wilcoxon rank sum test with continuity correction (non-parametric distribution). Categorical variables were compared by using the Pearson’s Chi-squared test with Yate’s continuity correction or the Fisher exact test for count data. Observed differences in clinical and genetic characteristics were confirmed by a general linear model by using the APOE polymorphism as confounding factor. Relative allelic frequencies were estimated by the gene-counting method [44]. The Hardy-Weinberg (HW) equilibrium was verified for each polymorphism. All statistical analyses were performed using the R Statistical Software Ver. 3.4.0 [45]. Tests in which the *p*-value was smaller than the Type I error rate α = 0.05 were declared significant.

## Results

During the enrolment period, 277 AD patients were screened for the inclusion in the study. Of these, 8 patients were excluded because they were younger than 65 years, 2 patients had an incomplete examination and 10 patients had severe comorbidity associated with cognitive impairment. Thus, the final population included 257 AD patients, 82 men (31.92%) and 175 women (68.09%) with a mean age of 80.21 ± 6.62 years (age range 65–97), of which 171 AD patients with delusions (66.54%) and 86 AD patients without delusions (33.46%).

Demographic and clinical characteristic of patients according to delusion symptom are reported in Table 1. AD patients with delusions showed a different social support network if compared with AD patients without delusions (19.88% living alone, 65.50% living with family, 14.62% institutionalized for AD patients with delusions vs. 2.33, 95.35, and 2.33% for AD patients without delusions; *p* < 0.001) and a higher number of concomitant treatments (6.60 ± 3.07 vs. 5.08 ± 3.12; *p* = 0.026). AD patients with delusions also showed a higher risk of malnutrition (MNA score 18.81 ± 5.77 vs. 20.98 ± 5.64; *p* = 0.011), a greater functional impairment, as measured by ADL (3.27 ± 1.98 vs. 3.89 ± 1.86; *p* = 0.025) and IADL (2.05 ± 2.39 vs. 2.87 ± 2.81; *p* = 0.037), a greater number of pressure sores (ESS 14.50 ± 3.29 vs. 15.80 ± 3.29; *p* = 0.008), and an higher degree of cognitive impairment as measured by MMSE (16.81 ± 5.92 vs. 18.33 ± 5.64; *p* = 0.017) and SPMSQ (5.29 ± 1.93 vs. 4.75 ± 1.87; *p* = 0.014), a worse neuropsychiatric condition (NPI score 35.57 ± 20.94 vs. 22.72 ± 17.04; *p* < 0.001), with an associated greater burden level (NPI-Distress score 15.71 ± 8.08 vs. 9.32 ± 5.96; *p* < 0.001).

**Table 1:** Demographic and clinical characteristics of Alzheimer’s disease (AD) patients according to the presence of delusion symptom

		AD-D	AD-noD	p	All
		(*n* = 171)	(*n* = 86)		(*N* = 257)
Sex^a^	(M/F)	55/116	27/59	0.999	82/175
	(Ratio)	0.47	0.46		0.47
Age	(Years)	79.84 ± 6.80	80.97 ± 6.22	0.254	80.21 ± 6.62
Body mass index	(Kg/m^2^)	26.81 ± 5.89	25.15 ± 6.03	0.106	26.30 ± 5.95
Educational level	(Years)	4.25 ± 3.83	4.26 ± 3.49	0.798	4.26 ± 3.72
Social support network^b^	Living alone	34 (19.88%)	2 (2.33%)	0.000	36 (14.01%)
	Living with family	112 (65.50%)	82 (95.35%)		194 (75.49%)
	Institutionalized	25 (14.62%)	2 (2.33%)		27 (10.51%)
Concomitant treatments	(No. of drugs)	6.60 ± 3.07	5.08 ± 3.12	0.026	6.15 ± 3.14
*Clinical characteristics*					
MNA		18.81 ± 5.77	20.98 ± 5.64	0.011	19.48 ± 5.81
ADL		3.27 ± 1.98	3.89 ± 1.86	0.025	3.47 ± 1.96
IADL		2.05 ± 2.39	2.87 ± 2.81	0.037	2.31 ± 2.55
CIRS-CI		2.74 ± 1.62	2.73 ± 1.74	0.870	2.74 ± 1.66
ESS		14.50 ± 3.29	15.80 ± 3.29	0.008	14.89 ± 3.33
SPMSQ		5.29 ± 1.93	4.75 ± 1.87	0.014	5.11 ± 1.93
Age at onset^c^	(Years)	78.03 ± 6.68	78.84 ± 6.16	0.143	78.02 ± 6.53
Disease duration	(Years)	2.23 ± 0.88	2.09 ± 0.94	0.226	2.18 ± 0 90
MMSE		16.81 ± 5.92	18.33 ± 5.64	0.017	17.32 ± 5.86
NPI		35.57 ± 20.94	22.72 ± 17.04	0.000	31.14 ± 20.57
NPI-Distress		15.71 ± 8.08	9.32 ± 5.96	0.000	13.58 ± 8.02
Delusion symptom duration	(Years)	2.99 ± 0.79	–	–	1.39 ± 1.18
Delusion symptom severity		6.38 ± 3.25	–	–	4.27 ± 4.01

Values of continuous variables are presented as mean ± standard deviation (SD)

^a^Fisher’s Exact Test for Count Data; ^b^Pearson’s Chi-squared test; ^c^Welch Two Sample t-test. All the other variables were compared by the Wilcoxon rank sum test with continuity correction

*AD-D* AD patients with delusions, *AD-noD* AD patients without delusions, *MNA* Mini Nutritional Assessment, *ADL* Activities of Daily Living, *IADL* Instrumental Activities of Daily Living, *CIRS-CI* Cumulative Illness Rating Scale Comorbidity Index, *ESS* Exton-Smith Scale, *SPMSQ* Short Portable Mental Status Questionnaire, *MMSE* Mini-Mental State Examination, *NPI* Neuropsychiatric Inventory

Prevalence of NPS according to delusion symptom is reported in Table 2. As compared with AD patients without delusions, AD patients with delusions showed a higher prevalence of hallucinations (not observed in AD patients without delusions), agitation/aggression (3.74 ± 3.57 vs. 0.46 ± 1.11; p < 0.001), anxiety (5.34 ± 4.44 vs. 3.42 ± 3.88; *p* < 0.001), euphoria (not observed in AD patients without delusions), disinhibition (not observed in AD patients without delusions), irritability/liability (4.17 ± 4.17 vs. 0.60 ± 1.28; p < 0.001), and aberrant motor activity (0.86 ± 2.11 vs. 0.43 ± 1.87; *p* = 0.029). In these patients, a trend towards a higher prevalence of apathy (5.75 ± 4.72 vs. 4.86 ± 4.75; *p* = 0.072) and sleep disturbance (5.52 ± 4.39 vs. 4.58 ± 4.45; *p* = 0.094) was also observed.

**Table 2:** Neuropsychiatric symptoms in Alzheimer’s disease (AD) patients according to to the presence of delusion symptoms

	AD-D	AD-noD	p	All
Hallucination	1.503 ± 3.265	–	–	1.000 ± 2.754
Agitation/aggression	3.737 ± 3.568	0.465 ± 1.113	0.000	2.642 ± 3.355
Depressed mood	5.643 ± 4.390	5.058 ± 4.517	0.314	5.447 ± 4.433
Anxiety	5.339 ± 4.444	3.419 ± 3.878	0.000	4.696 ± 4.351
Euphoria	0.491 ± 2.143	–	–	0.326 ± 1.761
Apathy	5.749 ± 4.718	4.860 ± 4.755	*0.072*	5.451 ± 4.739
Disinhibition	0.005 ± 0.076	–	–	0.003 ± 0.062
Irritability/liability	4.170 ± 4.174	0.604 ± 1.276	0.000	2.977 ± 3.866
Aberrant motor activity	0.859 ± 2.109	0.430 ± 1.875	0.029	0.716 ± 2.040
Sleep disturbance	5.518 ± 4.393	4.581 ± 4.454	*0.094*	5.203 ± 4.427
Eating disorder	2.848 ± 3.844	3.302 ± 4.424	0.615	3.000 ± 4.044

*AD-D* AD patients with delusions, *AD-noD* AD patients without delusions

**Fig. 1:**
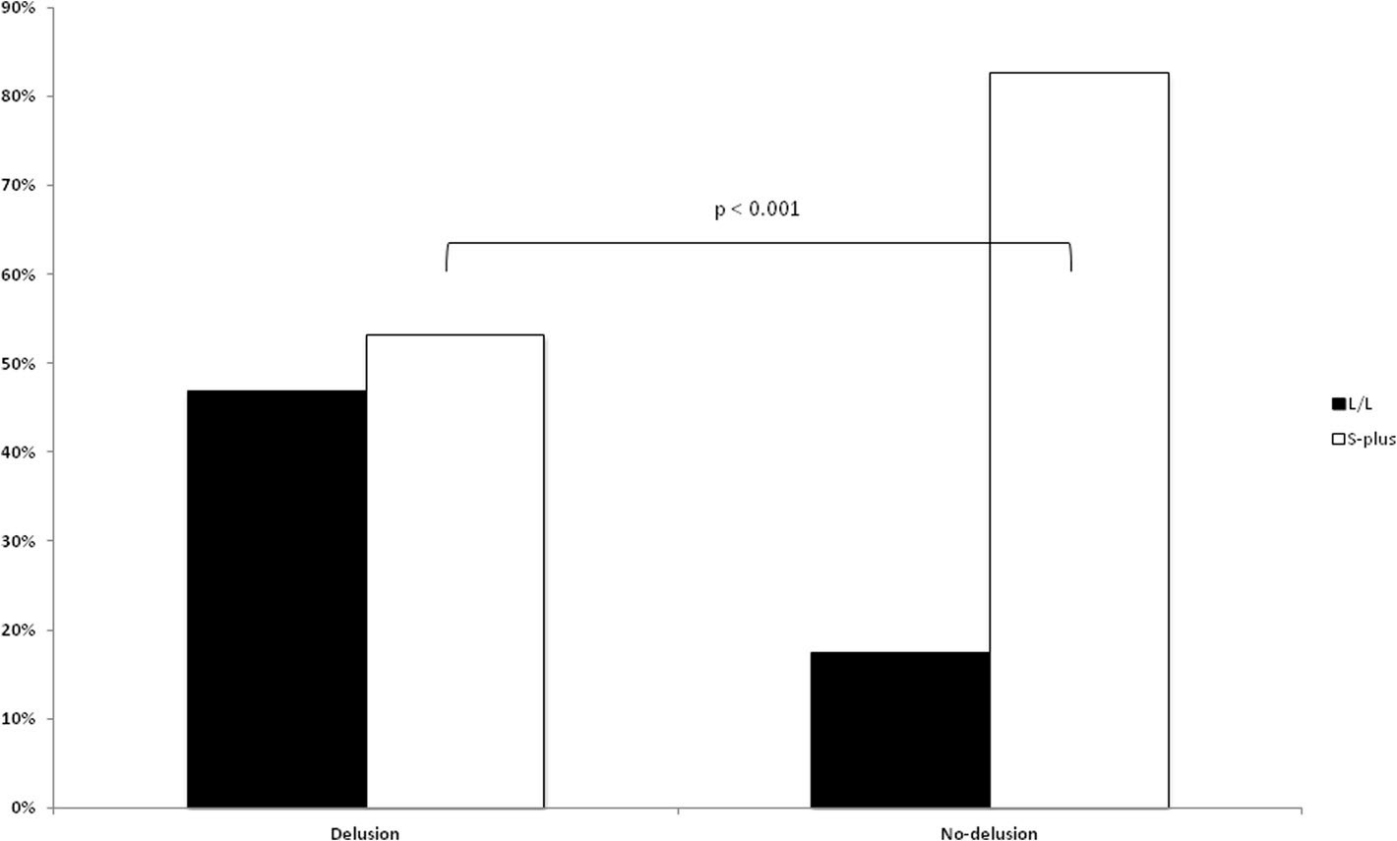
Carriers of 5-hydroxytriptamine transporter gene-linked polymorphic region (5-HTTLPR) genotypes L/L or L/S + S/S (S-plus) in Alzheimer’s disease patients with and without delusions

Prevalence of 5-HTTLPR genotypes and estimated allele frequencies, as well as those of the APOE polymorphism, are reported in Table 3. As compared with AD patients without delusions, the prevalence of heterozygotes 5-HTTLPR-L/S as well as homozygotes 5-HTTLPR-S/S was lower in AD patients with delusions [37.43% vs. 61.63%, p < 0.001; odds ratio (OR) = 0.226, 95% confidence interval (CI) = 0.111–0.459, and 20.93% vs. 15.79%, *p* = 0.003; OR = 0.281, 95% CI = 0.115–0.682, respectively). The prevalence of L/L vs. S plus (L/S + S/S) according to delusion symptom is graphically presented in Fig. 1. The estimated frequency of S allele also resulted lower in AD patients with delusions (0.345 vs. 0.517, *p* < 0.001; OR = 0.415, 95% CI = 0.166–1.038). In the analysis of the APOE polymorphism, no differences in genotype distribution according to delusion symptom was shown, despite a non significant lower frequency of the APOE ε2 allele in AD patients with delusions (0.032 vs. 0.070, *p* = 0.041; OR = 0.415, 95% CI = 0.166–1.038).

**Table 3:** Genetic characteristics of patients with Alzheimer’s disease (AD) according to the presence of delusion symptom

	AD-D		AD-noD		p	OR	(95% CI)	All	
5-HTTLPR L/L	80	(46.78%)	15	(17.44%)	Reference			95	(36.96%)
5-HTTLPR L/S	64	(37.43%)	53	(61.63%)	0.000	0.226	(0.111–0.459)	117	(45.53%)
5-HTTLPR S/S	27	(15.79%)	18	(20.93%)	0.003	0.281	(0.115–0.682)	45	(17.51%)
HWE	p = 0.029		p = 0.029					*p* = 0.439	
5-HTTLPR L	224	(0.655)	83	(0.483)	Reference			307	(0.597)
5-HTTLPR S	118	(0.345)	89	(0.517)	0.000	0.491	(0.332–0.726)	207	(0.403)
APOE ε2/ε2	–	–	–	–	–	–	–	–	–
APOE ε2/ε3	8	(4.68%)	7	(8.14%)	0.243	0.486	(0.150–1.586)	15	(5.84%)
APOE ε2/ε4	3	(1.75%)	5	(5.81%)	0.110	0.255	(0.046–1.286)	8	(3.11%)
APOE ε3/ε3	120	(70.18%)	51	(59.30%)	Reference			171	(66.54%)
APOE ε3/ε4	39	(22.81%)	21	(24.42%)	0.518	0.789	(0.405–1.544)	60	(23.35%)
APOE ε4/ε4	1	(0.58%)	2	(2.33%)	0.220	0.213	(0.007–3.081)	3	(1.17%)
HWE	*p* = 0.444		*p* = 0.223					*p* = 0.057	
APOE ε2	11	(0.032)	12	(0.070)	0.041	0.415	(0.166–1.038)	23	(0.045)
APOE ε3	287	(0.839)	130	(0.756)	Reference			417	(0.811)
APOE ε4	44	(0.129)	30	(0.174)	0.138	0.664	(0.388–1.139)	74	(0.144)

*AD-D* AD patients with delusions, *AD-noD* AD patients without delusions, *OR* odds ratio, *CI* confidence interval, *5-HTTLPR* 5-hydroxytriptamine transporter gene-linked polymorphic region, *APOE* apolipoprotein E

The APOE-adjusted associations of S-plus carriers with clinical characteristics according to delusion symptom are reported in Table 4 and Additional file 1: Figure S1. S-plus carriers have low disease duration in the overall sample (*p* = 0.011; OR = 0.740, 95% CI = 0.587–0.933), as well as in AD patients with delusions (*p* = 0.005; OR = 0.680, 95% CI = 0.522–0.886) (Additional file 1: Figure S1, panel A). In these patients, a trend toward higher NPI-Distress was also suggested (*p* = 0.074; OR = 9.564, 95% CI = 8.161 × 10^− 1^-1.121 × 10^2^) (Additional file 1: Figure S1, Panel B). The APOE-adjusted association of S-plus carriers with NPS according to delusion symptom is reported in Table 5 and Additional file 2: Figure S2. S-plus carriers had higher aberrant motor activity in the overall sample (*p* = 0.050; OR = 1.692, 95% CI = 1.004–2.850), as well as in AD patients with delusions (*p* = 0.013; OR = 2.257, 95% CI = 1.195–4.260) (Additional file 2: Figure S2, Panel A). In these patients, a trend toward higher irritability/liability was also suggested (*p* = 0.054; OR = 3.520, 95% CI = 0.988–12.500) (Additional file 2: Figure S2, Panel B).

**Table 4:** Apolipoprotein E-adjusted estimates of the association of 5-hydroxytriptamine transporter gene-linked polymorphic region (5-HTTLPR) S-plus carriers with clinical characteristics in patients with Alzheimer’s disease (AD) according to to the presence of delusion symptom

	All			AD-D			AD-noD		
	p	OR	(95%CI)	p	OR	(95%CI)	p	OR	(95%CI)
MNA	0.464	1.892	(3.449 × 10^−1^ - 1.038 × 10^1^)	0.884	1.157	(0.163–8.205)	0.830	0.607	(5.772 × 10^−3^ - 6.375 × 10^1^)
ADL	0.930	0.978	(0.582–1.640)	0.702	0.886	(0.478–1.644)	0.200	0.449	(0.132–1.528)
IADL	0.349	1.383	(0.703–2.720)	0.420	1.356	(0.644–2.855)	0.367	0.418	(0.064–2.745)
CIRS-CI	0.979	1.007	(0.582–1.750)	0.605	0.848	(0.454–1.583)	0.154	3.162	(0.667–15.002)
ESS	0.220	1.810	(0.709–4.600)	0.400	1.600	(0.543–4.715)	0.310	0.265	(0.021–3.414)
SPMSQ	0.993	1.000	(0.611–1.650)	0.943	1.022	(0.570–1.830)	0.140	2.310	(0.770–6.931)
Age at onset	0.706	1.380	(0.259–7.350)	0.539	0.528	(0.069–4.031)	0.320	6.104	(1.748 × 10^−1^ - 2.132 × 10^2^)
Disease duration	0.011	0.740	(0.587–0.933)	0.005	0.680	(0.522–0.886)	0.700	1.116	(0.640–1.947)
MMSE	0.996	1.000	(0.223–4.510)	0.888	0.880	(0.148–5.215)	0.210	0.121	(4.464 × 10^−3^ - 3.290)
NPI	0.950	0.854	(4.530 × 10^−3^ - 1.610 × 10^2^)	0.235	4.796 × 10^1^	(8.237 × 10^−2^ - 2.792 × 10^4^)	0.420	5.490 × 10^1^	(3.419 × 10^− 3^ - 8.817 × 10^5^)
NPI-Distress	0.870	1.180	(0.153–9.150)	*0.074*	*9.564*	*(8.161 × 10* ^*−1*^ *- 1.121 × 10* ^*2*^ *)*	0.234	7.960	(2.282 × 10^−1^ - 2.362 × 10^2^)

*AD-D* AD patients with delusions, *AD-noD* AD patients without delusions, *OR* odds ratio, *CI* confidence interval, *MNA* Mini Nutritional Assessment, *ADL* Activities of Daily Living, *IADL* Instrumental Activities of Daily Living, *CIRS-CI* Cumulative Illness Rating Scale Comorbidity Index, *ESS* Exton-Smith Scale, *SPMSQ* Short Portable Mental Status Questionnaire, *MMSE* Mini-Mental State Examination, *NPI* Neuropsychiatric Inventory

**Table 5:** Apolipoprotein E-adjusted estimates of the association of 5-hydroxytriptamine transporter gene-linked polymorphic region (5-HTTLPR) S-plus carriers with neuropsychiatric symptoms in patients with Alzheimer’s disease (AD) according to to the presence of delusion symptom

	All			AD-D			AD-noD		
	p	OR	(95%CI)	p	OR	(95%CI)	p	OR	(95%CI)
Hallucination	0.600	0.835	(0.412–1.700)	0.480	1.436	(0.529–3.900)	–	–	–
Agitation/aggression	0.504	0.745	(0.314–1.770)	0.139	2.280	(0.769–6.770)	0.160	1.586	(0.833–3.020)
Depressed mood	0.580	0.727	(0.236–2.240)	0.561	0.674	(0.178–2.540)	0.611	1.960	(1.480 × 10^−1^ - 2.610 × 10^1^)
Anxiety	0.216	0.498	(0.166–1.500)	0.582	0.681	(0.174–2.670)	0.610	1.730	(2.110 × 10^−1^ - 1.427 × 10^1^)
Euphoria	0.970	0.992	(0.631–1.560)	0.570	1.210	(0.628–2.330)	–	–	–
Apathy	0.968	1.025	(0.306–3.440)	0.742	1.270	(0.306–5.270)	0.690	1.780	(1.070 × 10^−1^ - 2.950 × 10^1^)
Disinhibition	0.490	1.006	(0.990–1.020)	0.390	1.010	(0.987–1.030)	–	–	–
Irritability/liability	0.883	0.928	(0.344–2.510)	*0.054*	*3.520*	*(0.988–12.500)*	0.280	1.505	(0.720–3.150)
Aberrant motor activity	0.050	1.692	(1.004–2.850)	0.013	2.257	(1.195–4.260)	0.550	1.407	(0.461–4.300)
Sleep disturbances	0.889	0.923	(0.299–2.850)	0.731	1.266	(0.330–4.860)	0.923	1.130	(8.860 × 10^−2^ - 1.450 × 10^1^)
Eating disorder	0.464	1.476	(0.521–4.180)	0.801	1.161	(0.364–3.710)	0.514	2.380	(1.780 × 10^−1^ - 3.186 × 10^1^)

*AD-D* AD patients with delusions, *AD-noD* AD patients without delusions, *OR* odds ratio, *CI* confidence interval;

## Discussion

In the present study, investigating the role of 5-HTTLPR in delusion symptom in a large cohort of 257 AD patients, we found a great difference in the distribution of the S-plus carriers (5-HTTLPR-L/S + 5-HTTLPR-S/S genotypes), suggesting an important role of serotonin in delusion symptom physiology. In fact, assuming the L/L genotype as the reference in the statistical analysis, the main finding of the present study was that S-plus carriers were strongly underrepresented in AD with delusions suggesting a possible protective role. Other NPS in AD patients such as apathy and sleep disturbance, which are also associated with dysfunction of the serotonin system, also trended towards a significance difference between the two genotyped cohorts. Furthermore, AD patients with delusions showed a higher prevalence of hallucinations, not observed in AD patients without delusions and also linked to serotonin system dysfunction. Estimating the role of 5-HTTLPR with the other clinical variables, particularly NPS, only minor effects were observed. In fact, 5-HTTLPR S-plus carriers mainly influenced disease duration (lower) and aberrant motor activity (higher). We also adjusted the analyses for the APOE polymorphism, the most important genetic factor influencing risk for AD, thus validating the quality of the present analysis.

In previous studies, the findings on the association of 5HTTLPR polymorphism with delusions in AD were contrasting [17–23]. In particular, a recent study showed that the homozygosis for the 5-HTTLPR-L/L genotype and lower MMSE scores were associated with an increased risk of delusion in DLB, with important implications regarding the mechanisms underlying this symptom across the AD/DLB/PD-dementia spectrum [24]. Similarly to our results, Sweet and colleagues reported an association between the L allele of the 5-HTTLPR polymorphic region with psychosis, as measured using the Empirical Behavioural Pathology in Alzheimer’s Disease [17]. However, Rocchi and colleagues did not find an association, using the NPI to investigate 135 Italian AD patients [18]. We can make a comparison with these two studies, because these investigators combined delusions and hallucinations to assess ‘psychosis’. In contrast to our results, Borroni and colleagues found that S allele carriers of 5HTTLPR had increased risk for the development of psychosis [19].

Among underlying mechanisms possibly explaining the association between 5-HTTLPR polymorphism and delusions in AD, it is well known that the serotonin system is important in the regulation of memory and thus might be associated with the disease [46, 47]. Serotonin has also been suggested to be responsible for a significant portion of the behavioural aspects of AD. The hypothesis is that transcriptional activity associated to the 5-HTTLPR-L allele is twice that of the S-allele. Thus, the S promoter allelic variant is linked to reduced 5-HTT mRNA expression, resulting in less serotonin reuptake than with the L-allelic variant and an increase of extraneuronal serotonin, contributing to the protective role in AD [48]. The specific mechanism by which the 5-HTTLPR may influence serotonin transmission is not known, but the difference in transcription rate between the two alleles may lead to differences in the number of serotonin reuptake sites and consequently affect serotonin levels [15, 16]. In the present study, AD patients with delusions showed a higher prevalence of hallucinations, not observed in AD patients without delusions. Given the central role of the serotonin system also in hallucinations, a genetic factor related to serotonin metabolism might exert a protective role on the clinical expression of psychotic clusters in AD. On the other hand, also nongenetic factors could influence the development of NPS in AD, i.e., these two cohorts of AD patients (i.e. with or without delusions) also had different social networks (or extent of said networks), and social network size modified the association between AD neuropathology and clinical symptoms of the disease [49]. Social network size may be related to reserve capacity capable of reducing the likelihood that the disease pathology will be clinically expressed [49]. Moreover, there was no influence of sex on the present findings. In fact, gender differences may potentially impact mortality in AD [50], APOE e4 allele effects on amyloid pathology [51], and the development of NPS [11]. However, the two present cohorts of AD patients with and without delusions had similar male/female ratios.

Furthermore, the effects of a 5-HTTLPR gene polymorphism on a functionally related but distinct receptor (5-HT1A receptor) were also evidenced. A study of positron emission tomography imaging has demonstrated the 5-HTTLPR-S/S genotype to be associated with a potential lower serotonin receptor 5HT_1A_ binding than the 5-HTTLPR-L/L genotype [52]. Mechanistically, the lower transcriptional efficiency associated with the S allele of the 5-HTTLPR may lead to decreased 5-HTT function, which in turn may lead to a lifelong increase in 5-HT tone, which may in turn desensitize and downregulate 5-HT1A receptors. Several studies have suggested an association between the 5-HTT genotype and physiological reactivity to acute stressors [53]. According to the plasticity hypothesis, the 5-HTTLPR-S allele has been identified as a genetic invulnerability factor to the negative effects of a preponderance of stressful life events, and also disproportionally benefit from a preponderance of positive environmental influences, which was not found for 5-HTTLPR-L/L homozygotes [54].

In light of these theories, the role of association studies on the neuropharmacology of serotonin in NPS in AD is still not clearly defined. Recently, a study suggested that serotonin receptor inverse agonists of 5-HT_2A_, such as pimavanserin, might have therapeutic benefits in the treatment of psychosis in AD patients [55], without negative effects on cognition and with no effect on dopamine receptors. Moreover, randomized clinical trials (RCTs) demonstrated that atypical antipsychotic agents which influence serotonin neurotransmission decreased psychosis in AD patients [56, 57]. Furthermore, it has been reported that psychotic disturbances may benefit from selective serotonin reuptake inhibitor treatment in patients with dementia [58]. Thus, a genetic factor related to serotonin metabolism might exert a protective role on the clinical expression of NPS clusters in AD.

We must acknowledge some limitations for the present report. First of all, as a general issue, the present candidate gene approach may have a greater propensity to yield false positives, and to investigate complex traits might be indicate to use a GWAS approach, given also that is highly likely that psychosis in AD is polygenic [12]. Moreover, the study population comprised only Caucasian patients recruited in a single centre, therefore, it could be possible that our findings may not be applicable in other populations. However, the S-plus carriers strongly underrepresented in our AD patients with delusions was a finding also confirmed by the great difference between the observed genotype frequencies in respect to the expected HW frequencies in the two subgroups of patients. In fact, this difference was not present in the entire cohort, being in HWE. This condition is synonymous of strong genetic association with the observed phenotype. It is clear that sampling bias influencing the HW equilibrium or the observed association cannot be excluded, including the relative small number of patients. However, the restricted confidence intervals given back from the analyses certified the sufficient size of the investigated sample.

## Conclusions

The present findings suggested that serotonin transporter gene may be associated with delusions in AD patients, providing important mechanistic information to boost the development of new treatment approaches and the prioritization and design of RCTs to manage NPS across the AD/DLB/PD-dementia spectrum.

## Additional files

Additional file 1: **Figure S1** The clinical parameters affected by 5-hydroxytriptamine transporter gene-linked polymorphic region (5-HTTLPR) carrier status disease duration (A) and Neuropsychiatric Inventory (NPI)-Distress (B) in the whole sample (left graphs), Alzheimer’s disease (AD) patients with delusions (central graphs), and AD patients without delusions (right graphs). Median, interquartile range, and extremes are presented. (TIF 86 kb)

Additional file 2: **Figure S2** The neuropsychiatric symptoms affected by 5-hydroxytriptamine transporter gene-linked polymorphic region (5-HTTLPR) carrier status aberrant motor activity (A) and irritability/liability (B) in the whole sample (left graphs), Alzheimer’s disease (AD) patients with delusions (central graphs), and AD patients without delusions (right graphs). Median, interquartile range, and extremes are presented. (TIF 84 kb)
