# Supplementary figures and images for: Hydroxytryptamine transporter gene-linked polymorphic region (5HTTLPR) is associated with delusions in Alzheimer’s disease
Source: Transl Neurodegener. 2019 Feb 1;8:4. doi: 10.1186/s40035-019-0144-1 (PMC6357440; doi:10.1186/s40035-019-0144-1)

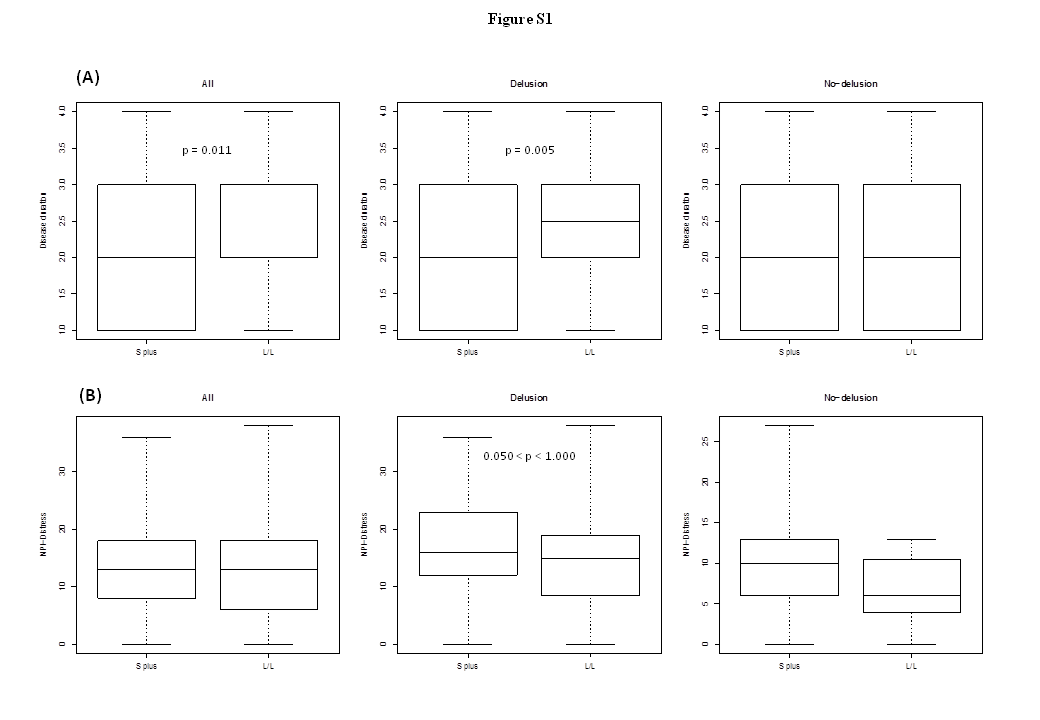

Supplement: Supplementary file 1 — Figure S1 The clinical parameters affected by 5-hydroxytriptamine transporter gene-linked polymorphic region (5-HTTLPR) carrier status disease duration (A) and Neuropsychiatric Inventory (NPI)-Distress (B) in the whole sample (left graphs), Alzheimer’s disease (AD) patients with delusions (central graphs), and AD patients without delusions (right graphs). Median, interquartile range, and extremes are presented. (TIF 86 kb) [file 40035_2019_144_MOESM1_ESM.tif]

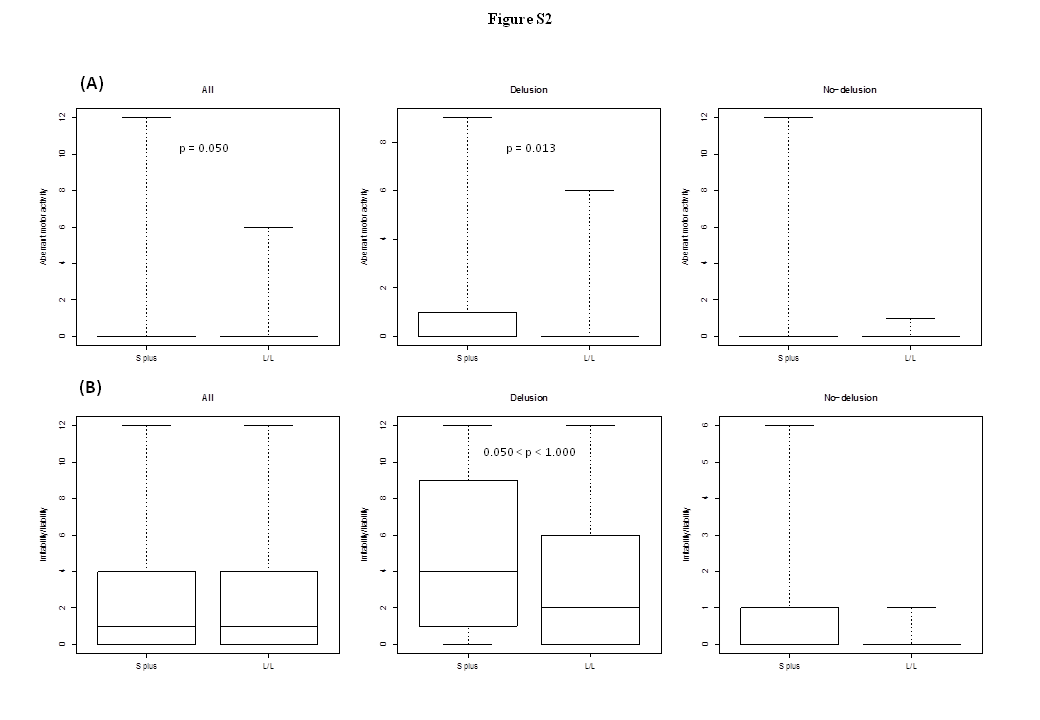

Supplement: Supplementary file 2 — Figure S2 The neuropsychiatric symptoms affected by 5-hydroxytriptamine transporter gene-linked polymorphic region (5-HTTLPR) carrier status aberrant motor activity (A) and irritability/liability (B) in the whole sample (left graphs), Alzheimer’s disease (AD) patients with delusions (central graphs), and AD patients without delusions (right graphs). Median, interquartile range, and extremes are presented. (TIF 84 kb) [file 40035_2019_144_MOESM2_ESM.tif]
